# Pressão Dipper ou não Dipper na Doença Pulmonar Obstrutiva Crônica: Eis a Questão!

**DOI:** 10.36660/abc.20201379

**Published:** 2021-02-19

**Authors:** Sofia F. Furlan, Luciano F. Drager

**Affiliations:** 1 Unidade de HipertensãoInstituto do CoraçãoHCFMUSPSão PauloSPBrasilUnidade de Hipertensão do Instituto do Coração (InCor) – HCFMUSP, São Paulo, SP - Brasil

**Keywords:** Doença Pulmonar Obstrutiva Crônica, Doenças Cardiovasculares, Monitoramento, Dipper, Não Dipper, Prognóstico, Qualidade de Vida

A doença pulmonar obstrutiva crônica (DPOC) é um problema grave de saúde mundial.^1^ É caracterizada como uma doença pulmonar inflamatória crônica que causa obstrução do fluxo de ar dos pulmões. É tipicamente causada pela exposição de longo prazo à fumaça do cigarro, mas a poluição do ar doméstico, as partículas ambientais, o ozônio e as partículas ocupacionais, incluindo poeira de carvão, também contribuem para a DPOC.^2^ De acordo com a Carga Global de Doenças, 544,9 milhões de pessoas ao redor do mundo apresentavam uma doença respiratória crônica em 2017, o que representava um aumento de 39,8% em comparação com 1990.^1^ Entre as doenças respiratórias, a DPOC permaneceu na condição específica de doença mais prevalente, globalmente, em 2017, respondendo por 55,1% da prevalência de doenças respiratórias crônicas entre homens e 54,8% entre mulheres.^1^ Um fato de maior importância é que a maioria dos óbitos atribuíveis a doenças respiratórias crônicas e dos anos de vida ajustados por incapacidade foram devidos à DPOC.^1^

Evidências consistentes indicam que a DPOC está associada ao aumento do risco cardiovascular, que é uma importante causa de morte em pacientes com DPOC.^3,4^ A inflamação sistêmica, a hipoxia crônica, a ativação simpática, a hiperinsuflação pulmonar, a eritrocitose secundária e a perda da superfície vascular pulmonar são responsáveis por aumentar o índice de condições como hipertensão pulmonar, disfunção ventricular direita, arritmias, doença coronariana isquêmica, entre outras.^3^ Mais recentemente, a associação entre DPOC e hipertensão tem ganhado cada vez mais atenção. Em uma coorte dinamarquesa de mais de 70.000 pacientes com DPOC, 47,6% dos pacientes tinham hipertensão (a comorbidade mais comum nestes pacientes).^5^ Embora não esteja claro se a DPOC aumenta a incidência de hipertensão, a pressão arterial (PA) não controlada está associada a um mau prognóstico em pacientes com DPOC.^6^ Estes achados abrem caminho para uma caracterização adicional do impacto da DPOC na variabilidade da PA.

Nesta edição dos *Arquivos Brasileiros de Cardiologia*,^7^os autores realizaram um interessante estudo transversal para investigar as associações entre o padrão pressórico não-dipper, que é um marcador de inflamação subclínica, rigidez arterial e qualidade de vida, em 142 pacientes adultos com DPOC. A DPOC foi definida por meio da espirometria e características clínicas sugestivas. Como esperado, todos os pacientes foram classificados como dipper ou não-dipper pelo monitoramento ambulatorial de PA de 24 horas (MAPA). Além disso, os autores avaliaram os parâmetros de rigidez arterial usando um dispositivo validado, calculando o índice de aumento e a velocidade da onda de pulso. A qualidade de vida foi avaliada por duas escalas, Questionário Respiratório de Saint George e a Escala de Qualidade de Vida Euro (EQ-5D). O primeiro é um questionário padronizado auto-aplicado para doenças das vias aéreas, dividido em três subescalas: sintomas (oito itens), atividade (16 itens) e impactos (26 itens). Para cada subescala e para o questionário geral, os escores variam de zero (sem comprometimento) a 100 (comprometimento máximo). A EQ-5D é um instrumento não específico de doença para descrever e avaliar a qualidade de vida relacionada à saúde. É notável que a intenção inicial dos autores ao usar essas duas escalas não estava clara. Os autores encontraram uma porcentagem muito alta de padrão pressórico não-dipper (< 10% de redução na PA durante o sono em comparação com o período de vigília) em pacientes com DPOC, ou seja, 76,1% (n = 108). Conforme descrito anteriormente em outras investigações, foram encontrados valores mais altos do índice de aumento naqueles com padrão pressórico não-dipper. É interessante que a qualidade de vida (medida pela EQ-5D) foi inferior em pacientes com DPOC que apresentavam o padrão pressórico não-dipper. De modo consistente, o Questionário Respiratório de Saint George revelou valores mais elevados (menor qualidade de vida) ao comparar nos padrões não-dipper e dipper. Na regressão logística multivariada, os participantes com padrão pressórico não-dipper apresentaram maiores valores de proteína C reativa (12%), índice de aumento (5,7%) e maior pontuação total no Questionário Respiratório de Saint George (2,1%), em comparação com o grupo de referência (padrão pressórico dipper). A EQ-5D não foi independentemente associada ao padrão pressórico não-dipper. Além disso, a frequência do padrão pressórico não-dipper aumentou paralelamente ao aumento do número de pessoas que habitavam o domicílio (33%).

O estudo realizado por Askin et al.^7^ tem mérito por abordar não somente a MAPA na DPOC, mas também as interfaces potenciais nesta associação. Três quartos dos pacientes com DPOC apresentaram padrão pressórico não-dipper, uma taxa comparável a outras condições crônicas, como diabetes e doença renal crônica.^8,9^ A associação independente do padrão não-dipper com a inflamação subclínica pode ter as seguintes duas implicações potenciais: 1) A inflamação pode ser um dos mecanismos potenciais do padrão pressórico não-dipper em pacientes com DPOC, mas o oposto também pode ser verdadeiro; 2) Esta combinação potencialmente denota um subgrupo de pacientes com DPOC com maior risco cardiovascular. A associação independente entre o número de pessoas que habitavam o domicílio e o padrão pressórico não-dipper é interessante e é potencialmente não específico para DPOC. Conforme especulado pelos autores, quanto maior o número de pessoas que habitam um local, maiores são os níveis de ansiedade (e possivelmente insônia) que, por sua vez, podem influenciar o padrão circadiano da PA. Apesar dos pontos fortes, é importante comentar algumas limitações para orientar possíveis investigações no futuro. Primeiro, esse desenho transversal torna impossível qualquer inferência sobre causalidade. Algumas associações (por exemplo, inflamação e padrão pressórico não-dipper) podem ser bidirecionais. Em segundo lugar, cerca de 50% dos pacientes com DPOC apresentavam diagnóstico formal de hipertensão. A inclusão detalhada dos efeitos do tratamento anti-hipertensivo seria necessária para melhorar a qualidade da análise multivariada. Terceiro, pacientes com o padrão pressórico não-dipper podem sofrer de distúrbios respiratórios do sono importantes e prevalentes, como apneia obstrutiva do sono (AOS).^10^ A síndrome de sobreposição, ou seja, a coexistência de DPOC e AOS, é relativamente comum e tem um impacto adicional no sistema cardiovascular, multiplicando o risco de morbimortalidade.^11,12^ Portanto, é concebível que a AOS seja um fator residual importante para explicar os principais resultados. Apesar da falta de dados detalhados sobre a MAPA na síndrome de sobreposição, evidências anteriores sobre a AOS mostraram que a PA diastólica atenuada e sistólica/diastólica com dipping inverso estão independentemente associadas com a AOS moderada a grave.

Em conclusão, a DPOC é potencialmente um “calouro” em termos de impacto no perfil de PA de 24 horas. Visto que a PA não-dipper possui significado prognóstico, são necessários estudos futuros com o objetivo de avaliar se o risco cardiovascular atribuído à DPOC é parcialmente mediado pelos parâmetros da MAPA.
