# Supplementary material for: Pressão Dipper ou não Dipper na Doença Pulmonar Obstrutiva Crônica: Eis a Questão!
Source: Arq Bras Cardiol. 2021 Feb 19;116(2):303–4. [Article in Portuguese] doi: 10.36660/abc.20201379 (PMC7909970; doi:10.36660/abc.20201379)
Supplement: Translation

# Translation: To Dip or not to Dip Blood Pressure in Chronic Obstructive Pulmonary Disease: That is the Question!

**Authors:** Sofia F. Furlan, Luciano F. Drager

**Affiliations:** 1 BrasilUnidade de Hipertensão do Instituto do Coração (InCor) – HCFMUSP, São Paulo, SP - Brasil

**Keywords:** Pulmonary Disease Chronic Obstructive, Cardiovascular Diseases, Monitoring, Dipper, Non-Dipper, Prognosis, Quality of Life

Chronic obstructive pulmonary disease (COPD) is a major health problem worldwide.^1^ It is characterized as a chronic inflammatory lung disease that causes obstructed airflow from the lungs. It is typically caused by long-term exposure to cigarette smoke, but household air pollution, ambient particulate matter, ozone, and occupational particulates, including coal dust, also contribute to COPD.^2^ According to the Global Burden of Disease, 544.9 million people worldwide had a chronic respiratory disease in 2017, which represents an increase of 39.8% compared with 1990.^1^ Among the respiratory diseases, COPD remained the most prevalent disease-specific condition in 2017, accounting for 55.1% of chronic respiratory disease prevalence among men and 54.8% among women globally.^1^ More importantly, most chronic respiratory disease-attributable deaths and disability-adjusted life years were due to COPD.^1^

Consistent evidence has indicated that COPD is associated with increased cardiovascular risk, which is an important cause of death in COPD patients.^3,4^ Systemic inflammation, chronic hypoxia, sympathetic activation, lung hyperinflation, secondary erythrocytosis, and loss of pulmonary vascular surface are responsible for increasing the rate of conditions such as pulmonary hypertension, right ventricular dysfunction, arrhythmias, ischemic coronary disease, among others.^3^ More recently, the association between COPD and hypertension has gained increasing attention. In a Danish cohort of more than 70,000 COPD patients, 47.6% of the patients had hypertension (the most common comorbidity in these patients).^5^ Although it is not clear whether COPD increases the incidence of hypertension, uncontrolled blood pressure (BP) is associated with poor prognosis in patients with COPD.^6^ These findings pave the way for additional characterization of the impact of COPD on BP variability.

In this issue of the *Arquivos Brasileiros de Cardiologia*,^7^the authors conducted an interesting cross-sectional study to investigate the associations between non-dipper BP patterns, which are markers of subclinical inflammation, arterial stiffness, and quality of life, in 142 adult patients with COPD. COPD was defined using spirometry and suggestive clinical features. As expected, all patients were classified as dippers or non-dippers by 24-hour ambulatory BP monitoring (ABPM). In addition, the authors assessed arterial stiffness parameters using a validated device calculating the augmentation index and pulse wave velocity. The quality of life was evaluated by two scales, the Saint George’ Respiratory Questionnaire and the Euro Quality of Life Scale (EQ-5D). The first one is a standardized self-administered airways disease-specific questionnaire divided into three subscales: symptoms (eight items), activity (16 items), and impacts (26 items). For each subscale and for the overall questionnaire, scores range from zero (no impairment) to 100 (maximum impairment). The EQ-5D is a non-disease specific instrument to describe and evaluate the health-related quality of life. It is noteworthy that the initial intention of the authors for using these two scales was not clear. The authors found a very high percentage of non-dipper BP patterns (< 10% reduction in BP during sleep compared with the waking period) in patients with COPD, namely, 76.1% (n = 108). As previously described in other investigations, higher values of augmentation index were found in those with the non-dipper BP profile. Interestingly, the quality of life (measured by the EQ-5D) was lower in patients with COPD who presented a non-dipping BP pattern. Consistently, the Saint George Respiratory Questionnaire revealed higher values (less quality of life) when comparing non-dippers and dippers. In the multivariate logistic regression, participants with the non-dipper BP pattern presented higher values of C-reactive protein (12%), augmentation index (5.7%), and a higher total score of the Saint George Questionnaire (2.1%), compared to the reference group (dipper BP pattern). EQ-5D was not independently associated with non-dipping BP pattern. Moreover, the frequency of the non-dipper pressure BP pattern increased in parallel to the increased number of people living in the household (33%).

The study conducted by Askin et al.^7^ has merit for addressing not only ABPM in COPD but also potential interfaces in this association. Three quarters of COPD patients presented non-dipping BP pattern, a rate comparable to other chronic conditions, such as diabetes and chronic kidney disease.^8,9^ The independent association of non-dipping BP with subclinical inflammation may have the following two potential implications: 1) Inflammation may be one of the potential mechanisms of non-dipping BP pattern in patients with COPD, but the opposite may also be true; 2) This combination potentially denotes a sub-group of patients with COPD with higher cardiovascular risk. The independent association between the number of people living in the household and the non-dipper BP pattern is interesting, and it is potentially not specific to COPD. As speculated by the authors, the higher the number of people living in a place, the higher the levels of anxiety (and possibly insomnia) that in turn may influence the circadian BP pattern. Despite the strengths, it is important to comment on some limitations to guide potential investigations in the future. First, this cross-sectional design prevents any inference about causality. Some associations (for instance, inflammation and non-dipping BP pattern) may be bidirectional. Second, around 50% of patients with COPD had a formal diagnosis of hypertension. Detailed inclusion of the effects of anti-hypertensive treatment would be necessary to improve the quality of the multivariate analysis. Third, patients with non-dipping BP may suffer from important and prevalent sleep-disordered breathing, such as obstructive sleep apnea (OSA).^10^ Overlap syndrome, i.e. the co-existence of both COPD and OSA, is relatively common, and it has an additional impact on cardiovascular system, multiplying the risk of morbidity and mortality.^11,12^ Therefore, it is conceivable that OSA is a major residual factor for explaining the main results. Despite the lack of detailed data on ABPM in overlap syndrome, previous evidence on OSA showed that diastolic attenuated and systolic/diastolic reverse dipping BP are independently associated with moderate to severe OSA.

In conclusion, COPD is potentially a “new kid in the block” in terms of impact on the 24-hour BP profile. Because non-dipping BP has prognostic significance, future studies aiming to evaluate whether the cardiovascular risk attributed to COPD is partially mediated by ABPM parameters are necessary.
